# Differences in Cortical Thickness in Schizophrenia Patients With and Without Auditory Verbal Hallucinations

**DOI:** 10.3389/fnmol.2022.845970

**Published:** 2022-05-12

**Authors:** Honghong Ren, Qianjin Wang, Chunwang Li, Zongchang Li, Jinguang Li, Lulin Dai, Min Dong, Jun Zhou, Jingqi He, Yanhui Liao, Ying He, Xiaogang Chen, Jinsong Tang

**Affiliations:** ^1^Department of Psychiatry, and National Clinical Research Center for Mental Disorders, The Second Xiangya Hospital of Central South University, Changsha, China; ^2^Hunan Key Laboratory of Psychiatry and Mental Health, Changsha, China; ^3^Department of Radiology, Hunan Children’s Hospital, Changsha, China; ^4^Guangdong Mental Health Center, Guangdong Provincial People’s Hospital, Guangdong Academy of Medical Sciences, Guangzhou, China; ^5^Department of Psychiatry, School of Medicine, Sir Run-Run Shaw Hospital, Zhejiang University, Hangzhou, China

**Keywords:** schizophrenia, auditory verbal hallucinations, orbitofrontal, cortical thickness, magnetic resonance imaging

## Abstract

Auditory verbal hallucinations (AVHs) are one of the most common and severe symptoms of schizophrenia (SCZ), but the neuroanatomical mechanisms underlying AVHs remain unclear. This study aimed to investigate whether persistent AVHs (pAVH) are associated with cortical thinning of certain brain regions in patients with SCZ. With the use of the 3T magnetic resonance imaging (MRI) technology, we acquired and analyzed data from 79 SCZ patients with pAVH (pAVH group), 60 SCZ patients without AVHs (non-AVH group), and 83 healthy controls (HC group). The severity of pAVH was assessed by the P3 hallucination items in the Positive and Negative Syndrome Scale (PANSS) and the Auditory Hallucinations Rating Scale (AHRS). Cortical thickness analysis was used to compare the region of interest (ROI) cortical thickness between the groups. The relationship between the severity of pAVH and cortical thickness was also explored. Compared with the non-AVH and HC groups, the pAVH group exhibited significantly reduced cortical thickness in the bilateral lateral orbitofrontal region (*p* < 0.0007, after Bonferroni correction); no significant difference was found between the non-AVH group and the HC group. The cortical thickness of the left lateral orbitofrontal cortex (P3: *r* = −0.44, *p* < 0.001; AHRS: *r* = −0.45, *p* < 0.001) and the right lateral orbitofrontal cortex (P3: *r* = −0.36, *p* = 0.002; AHRS: *r* = −0.33, *p* = 0.004) were negatively correlated with the severity of pAVH (after Bonferroni correction, *p* < 0.0125). Therefore, abnormal thickness of the bilateral lateral orbitofrontal cortices might be associated with pAVHs in SCZ patients.

## Introduction

Schizophrenia (SCZ) is a chronic debilitating psychiatric disorder characterized by positive symptoms, negative symptoms, and cognitive dysfunction (Khan et al., 2018). Auditory verbal hallucinations (AVHs), as a hallmark positive symptom, are an important diagnostic criterion for SCZ (Chen et al., 2015). Persistent AVHs (pAVH) are AVHs that persist for more than one year despite treatment with two different antipsychotics (González et al., 2006). Affecting approximately 60–80% of patients with SCZ (Sartorius et al., 1986), AVHs have brought a huge and continuous burden to the patients and are usually associated with social and occupational dysfunction as well as poor prognosis (Shergill et al., 1998; Goghari et al., 2013). Although antipsychotics can quickly reduce the frequency and severity of AVHs for most patients (Sommer et al., 2012), there are still 25–30% of SCZ cases being chronically resistant to conventional antipsychotics Liu et al. (2015).

Neuroimaging evidence has shown that AVHs are usually associated with abnormal structure and neuro-metabolism of frontal areas (Steinmann et al., 2014; Psomiades et al., 2018a). The frontal lobe, which is connected to the temporal lobe through the arcuate fasciculus, is a part of a key pathway in the language network (Glasser and Rilling, 2008) and plays a well-established functional role in auditory perception and language processing (Giraud et al., 2004). Previous studies have shown that patients with AVHs have lower gray matter volume (Kubera et al., 2014) and lower functional connectivity (Scheinost et al., 2019) in the frontal regions compared to patients without AVHs and healthy controls (HC). Furthermore, a series of studies using voxel-based morphometry (VBM) to investigate SCZ patients with AVHs showed structural changes in the extra-sensory regions other than the auditory cortex; specifically, SCZ patients with AVHs were found to have reduced gray matter volumes in the left insular cortex and the adjacent temporal pole (Shapleske et al., 2002), thalamus and cerebellum (Neckelmann et al., 2006), left transverse gyrus (Heschl gyrus, HG) (Modinos et al., 2013), left superior limbic gyrus, and dorsolateral prefrontal cortex (DLPFC) (Gaser et al., 2004).

Although both the surface area and thickness have been studied frequently, cortical thickness has so far received the most attention in developmental studies, with evidence suggesting that cortical thickness is less affected by individual differences in the surface area than the gray matter volume (Foland-Ross et al., 2016; Walhovd et al., 2017). Meanwhile, changes in cortical thickness were found to be more sensitive to disease status than changes in volume or surface area (Zuo et al., 2018). Cortical thickness specifically reflects the cellular structure of cortical neurons, neuropil, and neuroglia, including density and arrangement; regarding neuropathology, it affects the synaptogenesis, synaptic pruning, and myelination in the human brain (Selemon and Goldman-Rakic, 1999; Vidal-Pineiro et al., 2020).

Previous studies have found widespread declines in cortical thickness in patients with SCZ (Rodriguez-Perez et al., 2021), suggesting that cortical thinning might be a potential factor for the development of SCZ symptoms, including AVHs. However, only a small number of studies directly compared the cortical thickness between SCZ patients with and without AVHs, and they yielded inconsistent results. For instance, Cui et al. (2018) found reduced cortical thickness in the left middle temporal gyrus (MTG) in 115 patients with AVHs, as compared with 93 patients without AVH and 216 HCs. Mørch-Johnsen et al. (2017) found that the cortical thickness of the left HG was significantly reduced in 145 patients with AVHs, compared with 49 patients without AVHs and 279 HCs. However, Chen et al. (2015) reported that the cortex was significantly thinner in 18 first-episode SCZ patients with persistent AVHs regarding the right HG, compared with 31 first-episode SCZ patients who had never experienced AVHs and 50 HCs. However, the above studies only found significant results in the auditory regions of interest (ROI), and there have been few reports on areas other than the auditory areas. Some further previous studies using vertex-wise analysis found that AVHs were associated not only with cortical thinning in the language regions in the dominant hemisphere but also with increased cortical thickness in regions related to self-monitoring (van Swam et al., 2012; Cui et al., 2018). For instance, van Swam et al. (2012) found that patients with AVHs had increased cortical thickness in the cingulate cortex and parahippocampal gyrus, which play an important role in self-monitoring, compared with patients without AVHs. More specifically, self-monitoring showed that AVHs were produced by improper monitoring of internal speech production, leading to erroneous attributions to internal speech and external perception (Simons et al., 2017; Psomiades et al., 2018b). Oertel-Knöchel et al. (2013) investigated the vertex-wise cortical thickness among 31 patients with chronic SCZ, 29 first-degree relatives of SCZ patients, and 37 HCs, and found that the cortical thickness of SCZ patients was significantly lower than that of the HCs, most notably in the frontal and temporal lobes, the superior parietal lobe, and several limbic regions, with intermediate levels of cortical thickness in relatives. In view of the limited and inconsistent evidence available, the extent and nature of the impact of cortical thinning on AVHs remain to be explored.

In recent years, clinical practitioners and researchers have paid little attention to the study of pAVH in SCZ. The purpose of the present study was to investigate the relationship between cortical thickness and pAVH in SCZ patients. We explored the relationship between cortical thickness and severity of pAVH based on ROI analysis. Based on the results of previous studies, we hypothesized that the cortex thickness of the language-related brain regions might be thinner in SCZ patients with pAVH, compared to patients without AVHs and HCs. We also hypothesized that the cortex thickness in these areas might be associated with the severity of pAVH.

## Materials and Methods

### Participants

A total of 140 SCZ patients were recruited from the Psychiatric Clinic at the Second Xiangya Hospital of Central South University in China. Meanwhile, 85 HCs were recruited via advertisements from local communities. All the patients were diagnosed with SCZ according to the Diagnostic and Statistical Manual of Mental Disorders, Fifth Edition (DSM-5) by two trained senior psychiatrists using Mini-International Neuropsychiatric Interviews (Sheehan et al., 1998). The patients were included if they were: (1) Han Chinese aged between 16 and 45 years; (2) right-handed; (3) normal in hearing and intelligence; (4) with no history of substance abuse; and (5) with no history of major medical or neurological diseases or trauma. The patients were divided into two subgroups based on whether they had auditory hallucination. Through the assessment using the P3 subscale of the Positive and Negative Syndrome Scale (PANSS) (Benetti et al., 2015), 80 patients with a score > 3 (i.e., presence of pAVH) were assigned to the pAVH group, and 60 patients with a score of = 1 (i.e., absence of AVHs) were assigned to the non-AVH group (Andreasen et al., 2005). In the present study, SCZ patients with pAVH all met the diagnostic criteria for treatment-resistant SCZ, and the severity of their symptoms was significantly greater than that of the non-AVH group. Also, we required that SCZ patients with non-AVH never experienced AVHs throughout the course of their illness. The severity of pAVH was assessed using the Auditory Hallucinations Rating Scale (AHRS) for the pAVH group (Chen et al., 2015). None of the HCs met the diagnostic criteria for any DSM-5 mental disorder or had a history of early mental disorder or family history of mental illnesses. This study was approved by the Ethics Committee of the Second Xiangya Hospital, Central South University (No. S006, 2018), and was conducted in accordance with the Declaration of Helsinki. After being duly informed of the study details, including benefits and potential risks, all the participants provided written informed consent.

To minimize the effects of neuroleptic medications on brain structure, this study preferentially recruited patients who received second-generation antipsychotics (SGAs) and matched the medication dose between the two patient groups. Current and previous antipsychotic regimens (type, dose, and duration of use) were recorded. In the patient groups, 18 patients (12.9%) were treated with first-generation antipsychotics (FGAs) in combination with SGAs; The remaining 121 patients (87.1%) were treated with SGAs in combination with SGAs. Type of medicine: FGAs include Haloperidol, Sulpiride. SGAs include Aripiprazole, Clozapine, Olanzapine, Quetiapine, Risperidone, Paliperidone, Ziprasidone, and Amisulpride.

### Magnetic Resonance Imaging Data Acquisition

Magnetic resonance imaging (MRI) data of all participants were obtained within 24 h after enrollment. All the MRI data were acquired using a 3.0T MRI scanner (Siemens, Munich, Germany) with a 16-channel headcoil at the Magnetic Imaging Center of Hunan Children’s Hospital, Changsha, China. None of the patients used antipsychotic medications on the day of the MRI scan, and their medications were not adjusted prior to the scan. During the scanning, foam pads and earplugs were used to restrain head movement and attenuate noise. Anatomical T1-weighted MRI data were acquired using a 3D magnetization-prepared rapid acquisition gradient echo (3D MPRAGE) sequence with the following parameters: repetition time (TR) = 2,530 ms, echo time (TE) = 2.33 ms, flip angle = 7°, field of view = 256 × 256 mm, slice thickness = 1 mm, slice gap = 0 mm, and number of slices = 192. All data sets were visually inspected for distortion and motion artifacts. There were no major scanner upgrades or instrument replacements during the study period.

### Measurement of Cortical Thickness

All the MRI images were processed using the FreeSurfer software package (version 7.1.0),^1^ which has been described and validated in previous studies (Dale et al., 1999; Fischl et al., 1999). Image preprocessing included the following steps: motion correction, brain extraction, Talairach transformation, intensity correction, brain tissue segmentation, automatic topology correction, and surface deformation (Chen et al., 2015). The measurement of cortical thickness was obtained by reconstructing the boundary of gray matter and white matter and the surface of the cortex, and then calculating the distance between the surfaces at each point across the cortical mantle (Dale et al., 1999). The generated cortical surfaces were then carefully reviewed and manually corrected for technical accuracy. The thickness of each vertex on the cortical surface was mapped into a common spherical system, and the maps were smoothed using a Gaussian kernel with a full width at half maximum (FWHM) of 10 mm. The results were inspected and checked for quality according to the ENIGMA protocol.^2^ The cortex was parcellated into 68 regions according to the Desikan-Killiany atlas.

### Statistical Analysis

All statistical analyses were performed using SPSS 26.0 (SPSS Inc., Chicago, IL, United States). Prior to all the analyses, we tested the normality of each variable using the Kolmogorov-Smirnov test. Demographic and clinical data were compared between groups using Chi-squared test, one-way analysis of variance (ANOVA), or Mann-Whitney *U*-test, when appropriate. Univariate covariance analysis (ANCOVA) was used to compare the cortical thickness of brain regions between the three groups, with age, gender, education, and estimated total intracranial volume (eTIV) being covariates. *Post hoc* tests were then performed when significant differences were found in the above comparisons, using Bonferroni correction for ANCOVA (*p* < 0.05/68 = 0.0007) and multiple comparisons (*p* < 0.05/14 = 0.0036). Partial correlation analysis was used to investigate the relationship between the severity of AVHs and the cortical thickness of brain regions in the pAVH group, with age, gender, education, and eTIV being covariates (with Bonferroni correction, *p* < 0.05/2 × 2 = 0.0125). The threshold of statistical significance was set at *p* = 0.05 (two-tailed).

## Results

### Demographic and Clinical Characteristics

Data for 1 pAVH patient and 2 HCs were excluded due to loss to follow-up and contraindications to MRI, respectively. The final analysis included 79 pAVH patients, 60 non-AVH patients, and 83 HCs. The demographic and clinical characteristics are summarized in Table 1. There were no significant differences in age, gender, smoking status, and drinking status between the three groups. The education level of both the pAVH and non-AVH groups were significantly lower than that of the HC group, and the education level of the pAVH group was significantly lower than that of the non-AVH group (*post hoc* results for pAVH vs. non-AVH, *p* = 0.03; pAVH vs. HC, *p* < 0.001; non-AVH vs. HC, *p* = 0.01). The score of P3 hallucination item of PANSS for the pAVH group was significantly higher than that for the non-AVH group (*p* < 0.001). The pAVH and non-AVH groups did not differ in age at onset, illness duration, CPZ equivalent dosage, as well as PANSS-P, PANSS-N, PANSS-G, and PANSS-T scores (*p* > 0.05).

**TABLE 1:** Demographic and clinical characteristics of patients and healthy controls.

		Patients (*n* = 139)		Significance			
Characteristics	HC (*n* = 83)	pAVH (*n* = 79)	non-AVH (*n* = 60)	HC vs. pAVH vs. non-AVH	HC vs. non-AVH	HC vs. pAVH	pAVH vs. non-AVH
					
					*p*-value		
Gender (M/F), *n*	38/45	39/40	36/24	χ^2^ = 2.93 (0.23)	0.09	0.65	0.21
Age (y), (M ± SD)	26.80 ± 5.91	25.58 ± 5.51	26.95 ± 5.90	*F* = 1.26 (0.29)	1.00	0.55	0.50
Education (y), (M ± SD)	14.43 ± 2.65	11.68 ± 3.16	13.00 ± 2.82	*F* = 18.37 (<0.001)**	0.01*	<0.001**	0.03*
Smoker/non-smoker, n	12/71	12/67	14/46	χ^2^ = 2.26 (0.32)	0.17	0.90	0.22
Drinker/non-drinker, n	2/81	0/79	1/59	χ^2^ = 1.82 (0.40)	0.76	0.17	0.25
Age at onset (y), (M ± SD)	−	19.78 ± 4.11	21.17 ± 5.05	−	−	−	*U* = 1,983 (0.10)
Illness duration (y), (M ± SD)	−	7.16 ± 4.59	5.83 ± 3.81	−	−	−	*U* = 1,975 (0.09)
PANSS-P (M ± SD)	−	13.65 ± 2.83	13.00 ± 3.66	−	−	−	*U* = 2,069 (0.24)
PANSS-N (M ± SD)	−	15.08 ± 5.66	14.10 ± 7.31	−	−	−	*U* = 1,940 (0.09)
PANSS-G (M ± SD)	−	26.85 ± 6.56	27.02 ± 8.65	−	−	−	*U* = 2,168 (0.46)
P3 hallucination item of PANSS (M ± SD)	−	5.11 ± 0.87	1.00 ± 0.00	−	−	−	*U* = 0.00 (<0.001)**
PANSS-T (M ± SD)	−	54.87 ± 12.51	54.12 ± 16.65	−	−	−	*U* = 1,974 (0.09)
AHRS (M ± SD)	−	26.10 ± 4.46	−	−	−	−	−
CPZ equivalent (mg/d), (M ± SD)	−	654.04 ± 280.65	588.63 ± 332.58	−	−	−	*U* = 1,952 (0.08)

M, mean; SD, standard deviation; n, number; M/F, male/female; pAVH, persistent auditory verbal hallucinations; non-AVH, without auditory verbal hallucinations; HC, health control; PANSS, Positive and Negative Symptoms Scale; PANSS-T, PANSS total score; PANSS-P, PANSS positive score; PANSS-N, PANSS negative score; PANSS-G, PANSS general psychopathology score; AHRS: the Auditory Hallucinations Rating Scale; CPZ, chlorpromazine; -, not applicable; *p < 0.05; **p < 0.01.

### Differences in Cortical Thickness Between Groups

**FIGURE 1:**
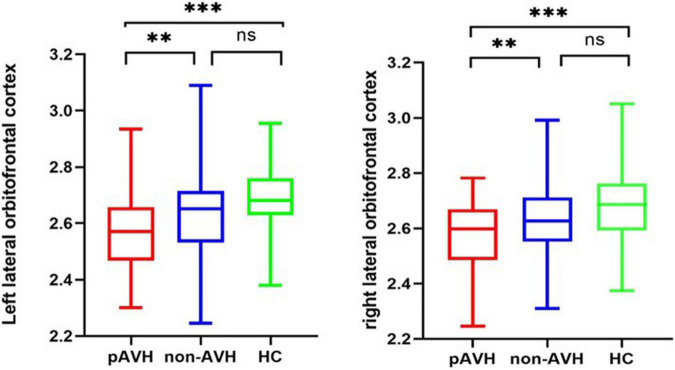
Cortical thickness of brain regions with significant differences between the three groups (with Bonferroni correction). **p < 0.01, ***p < 0.001; ns: not significant (*p* > 0.05); Bonferroni correction was used for ANCOVA (*p* < 0.0007) and multiple comparisons (*p* < 0.0036).

Inter-group ANCOVA with Bonferroni correction showed that the cortical thickness of the bilateral lateral orbitofrontal cortices was significantly lower in the pAVH group compared with the non-AVH and HC groups, while no differences were observed between the non-AVH and HC groups (see Table 2 and Figure 1). Although the level of education was different in the covariance analysis of the three groups, it did not affect the thickness of the bilateral lateral orbitofrontal cortices. Covariance analysis with education level as a covariate showed no difference in cortical thickness of bilateral lateral orbitofrontal cortices between the three groups (left lateral orbitofrontal cortex: *F* = 0.07, *p* = 0.79; right lateral orbitofrontal cortex: *F* = 1.59, *p* = 0.21). However, there were no differences in cortical thickness in other brain regions (*p* > 0.05) or differences without Bonferroni correction (all *p* > 0.0007) (see Supplementary Table 1).

**TABLE 2:** Brain regions with significant inter-group differences in cortical thickness.

	Cortical thickness (mm) M ± SD				Pairwise comparisons *P*-value		
Variable	pAVH	non-AVH	HC	ANCOVA	pAVH vs. non-AVH	pAVH vs. HC	non-AVH vs. HC
Left caudal middle frontal cortex	2.35 ± 0.14	2.39 ± 0.15	2.44 ± 0.13	*F* = 10.54, (<0.0007)	0.02	0.47	0.57
Left lateral orbitofrontal cortex	2.57 ± 0.13	2.63 ± 0.14	2.69 ± 0.11	*F* = 21.92, (<0.0007)	0.001*	<0.001*	0.01
Left pars opercularis cortex	2.32 ± 0.14	2.36 ± 0.16	2.42 ± 0.12	*F* = 11.74, (<0.0007)	0.26	<0.001*	0.01
Left pars orbitalis cortex	2.44 ± 0.17	2.42 ± 0.18	2.54 ± 0.17	*F* = 8.43, (0.0003)	1.00	0.001*	0.002
Left pars triangularis cortex	2.19 ± 0.14	2.21 ± 0.18	2.28 ± 0.15	*F* = 8,38, (0.0003)	0.36	<0.001*	0.05
Left rostral middle frontal cortex	2.14 ± 0.12	2.17 ± 0.11	2.22 ± 0.12	*F* = 10.57, (<0.0007)	0.20	<0.001*	0.03
Left superior frontal cortex	2.58 ± 0.14	2.60 ± 0.16	2.66 ± 0.14	*F* = 9.54, (0.0001)	0.32	<0.001*	0.03
Right caudal middle frontal cortex	2.38 ± 0.13	2.38 ± 0.16	2.45 ± 0.14	*F* = 8.29, (0.0003)	1.00	0.001*	0.003
Right lateral orbitofrontal cortex	2.57 ± 0.13	2.64 ± 0.14	2.68 ± 0.13	*F* = 19.63, (<0.0007)	<0.001*	<0.001*	0.11
Right medial orbitofrontal cortex	2.35 ± 0.14	2.40 ± 0.15	2.46 ± 0.13	*F* = 15.57, (<0.0007)	0.02	<0.001*	0.03
Right pars orbitalis cortex	2.48 ± 0.18	2.54 ± 0.16	2.62 ± 0.16	*F* = 20.09, (<0.0007)	0.007	<0.001*	0.007
Right pars triangularis cortex	2.22 ± 0.14	2.24 ± 0.15	2.33 ± 0.14	*F* = 14.78, (<0.0007)	0.19	<0.001*	0.003
Right rostral middle frontal cortex	2.12 ± 0.12	2.16 ± 0.13	2.19 ± 0.12	*F* = 9.42, (0.0001)	0.03	<0.001*	0.34
Right superior frontal cortex	2.53 ± 0.14	2.56 ± 0.16	2.64 ± 0.16	*F* = 12.28, (<0.0007)	0.15	<0.001*	0.02

M, mean; SD, standard deviation; mm, millimeter; ANCOVA, univariate covariance analysis; pAVH, persistent auditory verbal hallucinations; non-AVH, without auditory verbal hallucinations; HC, health control; Bonferroni correction was used for ANCOVA (p < 0.05/68 = 0.0007) and multiple comparisons (p < 0.05/14 = 0.0036). *p < 0.0036.

### Correlation Analysis Results

**FIGURE 2:**
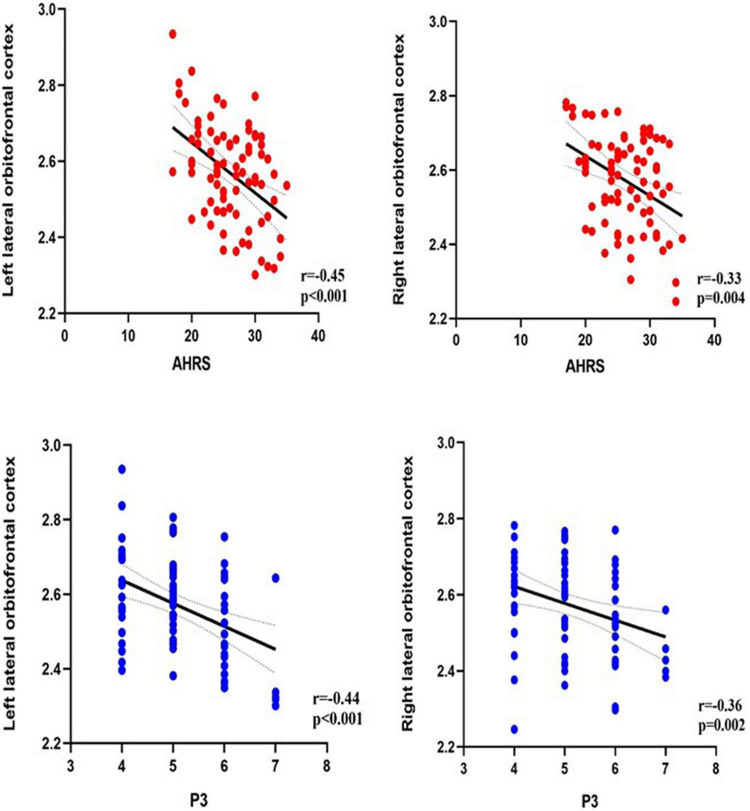
The relationship between the severity of pAVH (measured by P3 and AHRS) and cortical thickness of brain regions. P3, the P3 hallucination item in the Positive and Negative Syndrome Scale (PANSS); AHRS, the Auditory Hallucinations Rating Scale; Bonferroni correction (*p* < 0.0125).

Correlation analysis was performed between the brain regions with different cortical thicknesses identified by ANCOVA and the severity of pAVH to explore the potential relationship between the differences in cortical thickness and the severity of pAVH. The cortical thickness of the bilateral lateral orbitofrontal cortices was negatively correlated with the severity of pAVH (i.e., AHRS or P3 scores) (with Bonferroni correction, *p* < 0.0125; see Table 3 and Figure 2).

**TABLE 3:** Correlation between the cortical thickness of brain regions and the severity of pAVH.

	P3		AHRS	
Variable	*r*	*p*	*r*	*p*
Left lateral orbitofrontal cortex	−0.44	<0.001	−0.45	<0.001
Right lateral orbitofrontal cortex	−0.36	0.002	−0.33	0.004

*P3, the P3 hallucination item in the Positive and Negative Syndrome Scale (PANSS); AHRS, the Auditory Hallucinations Rating Scale; Bonferroni correction (p < 0.05/2× 2 = 0.0125).*

## Discussion

In the present study, we investigated the characteristics of cortical thickness in the ROI of SCZ patients with and without AVHs and its relationship with the severity of pAVH by using surface-based analysis. Our findings indicated that the brain regions with decreased cortical thickness in SCZ patients with pAVH were mainly in the frontal lobe, especially in bilateral lateral orbitofrontal cortices. We also found that the cortical thickness of the bilateral lateral orbitofrontal cortices in the pAVH group was negatively correlated with the severity of pAVH. In other words, thinner cortical thickness in these brain regions indicated higher severity of pAVH. The above findings are also consistent with our hypotheses.

We found that the cortical thickness of bilateral lateral orbitofrontal cortices decreased in the pAVH group compared with the non-AVH and HC groups. These areas belong to the orbital frontal cortex (OFC), which is involved in language, cognition, and emotional processing (Rubin et al., 2017; Gul and Yousaf, 2019). Previous studies have demonstrated that dysfunction of the OFC was associated with AVHs that are similar to those found in patients with SCZ. For example, a study using structural MRI (sMRI) found that the gray matter volume and cortical thickness of the OFC were lower in patients with SCZ and were negatively correlated with the score of the subscale for positive symptoms (including hallucinations) (Takayanagi et al., 2020). Previous studies using positron emission tomography (PET) and functional MRI (fMRI) also found abnormal activation of the OFC in SCZ patients with AVHs (Silbersweig et al., 1995; Ait Bentaleb et al., 2006). At present, the main theory of the neurocognitive model of AVHs is the neurocognitive action self-monitoring system (Frith et al., 2000), which holds that individuals erroneously perceive events that actually occur within as they are originated from outside. Patients with SCZ, especially those with AVHs, may have self-monitoring dysfunction (Waters et al., 2012), whereby they are unable to recognize events occurring within their minds and perceive them as events that have actually happened externally. Therefore, the OFC may play a key role in the self-monitoring system, which mediates the occurrence and development of AVHs.

Previous studies have also reported structural abnormalities in other brain regions, such as the temporal (Sun et al., 2009; Chen et al., 2015) and insular cortices (O’Daly et al., 2007); however, we did not find any alterations in the cortical thickness of these regions. Similarly, there was no difference in cortical thickness between the non-AVH group and the HC group in this study, while some studies found varying degrees of difference between the two groups (Köse et al., 2018). The inconsistent results might be attributed to methodological differences and heterogeneity in samples of SCZ. Most previous studies used voxel-based morphological (VBM) analysis, which focuses on structural changes in gray matter volume or density rather than cortical thickness. To the best of our knowledge, the samples of SCZ patients with AVHs were relatively small previous studies assessing cortical thickness (van Swam et al., 2012; Chen et al., 2015). Furthermore, SCZ patients present with a variety of clinical symptoms and different durations of illness. Evidence has demonstrated that patients with SCZ have cortical thinning over time throughout the course of the disease (van Haren et al., 2011), suggesting that the chronicity of the disease might be an important contributor to cortical thinning. In addition, the results of the present study are also indirectly supported by the results of previous studies on white matter volume, especially the studies involving arcuate fasciculus. The arcuate fasciculus, as it is known, is a bundle of nerve fibers connecting the frontal and temporal lobes within each cerebral hemisphere by passing dorsally beneath the parietal lobe, and is widely considered as a critical tract for language processing (Glasser and Rilling, 2008; Ivanova et al., 2021). For example, Salisbury et al. (2021) found that in the first psychotic episode, AVHs were more robustly associated with the microstructural deficits of the arcuate fasciculus and interhemispheric auditory fibers in the left hemisphere. Furthermore, a meta-analysis of studies involving diffusion tensor imaging of the arcuate fasciculus in patients with AVHs showed that the anisotropy score of the left arcuate fasciculus of the hallucinators was lower (Geoffroy et al., 2014). In this context, it was suggested that dysfunctional neuronal interconnections in white matter fibers between these regions are associated with the misperception of internal speech (Catani and ffytche, 2005). A disease-related cortical disorganization in the speech dominant hemisphere might affect white-matter connectivity in adjacent brain regions (van Swam et al., 2012).

There are some limitations to our study that need to be addressed. Firstly, the study is cross-sectional and follow-up visits are needed to further explore the development of symptoms and the prognosis, as well as the relationship between symptoms and cerebral cortical thickness. Secondly, all the patients were taking antipsychotics during the study period; although the results showed no difference in antipsychotic dosages between the two groups in the most recent year, the effect of drugs on cortical thickness could not be ruled out. In future works, the relationship between cortical thickness alterations and AVHs in first-episode treatment-naive patients with SCZ needs to be further explored. Finally, using illness duration as a covariance, covariance analysis found no difference in bilateral lateral orbitofrontal cortex thickness between the two patient groups. The possible reason is that the subjects in this study were all patients with chronic schizophrenia, and this study was cross-sectional, so it may not be able to highlight the influence of the illness duration on the disease. In future studies, follow-up or comparative studies involving first-episode patients, acute patients and chronic patients are needed to remedy this limitation.

## Conclusion

In summary, the present study showed that SCZ patients with pAVH exhibited decreased cortical thickness in the bilateral lateral orbitofrontal cortices, which were negatively correlated with the severity of pAVH. These findings suggest that anatomical defects in the frontal cortex, especially the bilateral lateral orbitofrontal cortices, may be associated with the pathogenesis of pAVH in patients with SCZ.

## Data Availability Statement

The datasets generated for this study are available on request to the corresponding author.

## Ethics Statement

The studies involving human participants were reviewed and approved by the Ethics Committee of the Second Xiangya Hospital, Central South University (No. S006, 2018). The patients/participants provided their written informed consent to participate in this study.

## Author Contributions

JT and XC designed and supervised the study. HR, JL, JH, LD, MD, and JZ collected the data. CL processed the scanning. HR, QW, JT, ZL, YL, and YH analyzed the data and interpreted the results. HR and QW first drafted the manuscript. JT, XC, ZL, and YL critically revised for important intellectual content. All authors revised and approved the final manuscript to be published.

## Conflict of Interest

The authors declare that the research was conducted in the absence of any commercial or financial relationships that could be construed as a potential conflict of interest.

## Publisher’s Note

All claims expressed in this article are solely those of the authors and do not necessarily represent those of their affiliated organizations, or those of the publisher, the editors and the reviewers. Any product that may be evaluated in this article, or claim that may be made by its manufacturer, is not guaranteed or endorsed by the publisher.

## Footnotes

1
http://surfer.nmr.mgh.harvard.edu

2
www.enigma.ini.usc.edu
