# Supplementary material for: Differences in Cortical Thickness in Schizophrenia Patients With and Without Auditory Verbal Hallucinations
Source: Front Mol Neurosci. 2022 May 12;15:845970. doi: 10.3389/fnmol.2022.845970 (PMC9135141; doi:10.3389/fnmol.2022.845970)
Supplement: Supplementary file 1 [file Table_1.docx]

Supplementary Table 1 Differences in cortical thickness of all brain regions between patients and healthy controls

| Variable | Cortical thickness(mm)  M±SD | | | ANCOVA |
| --- | --- | --- | --- | --- |
|  | AVH | non-AVH | HC |  |
| lh_bankssts_thickness | 2.13±0.18 | 2.14±0.17 | 2.17±0.17 | F=1.09, (0.34) |
| lh_caudalanteriorcingulate_thickness | 2.35±0.22 | 2.42±0.17 | 2.38±0.17 | F=3.61, (0.03) |
| lh_caudalmiddlefrontal_thickness | 2.35±0.14 | 2.39±0.15 | 2.44±0.13 | F=10.54, (0.000043) |
| lh_cuneus_thickness | 1.88±0.13 | 1.90±0.13 | 1.90±0.12 | F=0.83, (0.44) |
| lh_entorhinal_thickness | 3.27±0.26 | 3.29±0.28 | 3.33±0.25 | F=0.96, (0.38) |
| lh_fusiform_thickness | 2.68±0.11 | 2.73±0.12 | 2.75±0.10 | F=6.24, (0.002) |
| lh_inferiorparietal_thickness | 2.27±0.12 | 2.26±0.12 | 2.31±0.12 | F=3.44, (0.03) |
| lh_inferiortemporal_thickness | 2.56±0.15 | 2.60±0.15 | 2.62±0.14 | F=4.94, (0.008) |
| lh_isthmuscingulate_thickness | 2.26±0.15 | 2.26±0.14 | 2.31±0.13 | F=4.58, (0.01) |
| lh_lateraloccipital_thickness | 2.12±0.10 | 2.16±0.12 | 2.16±0.11 | F=4.36, (0.01) |
| lh_lateralorbitofrontal_thickness | 2.57±0.13 | 2.63±0.14 | 2.69±0.11 | F=21.92, (2.1762E-9) |
| lh_lingual_thickness | 2.01±0.11 | 2.05±0.13 | 2.05±0.11 | F=2.88, (0.06) |
| lh_medialorbitofrontal_thickness | 2.38±0.13 | 2.39±0.14 | 2.43±0.11 | F=6.10, (0.003) |
| lh_middletemporal_thickness | 2.46±0.17 | 2.49±0.14 | 2.49±0.16 | F=2.02, (0.14) |
| lh_parahippocampal_thickness | 2.66±0.22 | 2.73±0.26 | 2.75±0.25 | F=3.17, (0.04) |
| lh_paracentral_thickness | 2.45±0.15 | 2.44±0.15 | 2.46±0.15 | F=0.21, (0.81) |
| lh_parsopercularis_thickness | 2.32±0.14 | 2.36±0.16 | 2.42±0.12 | F=11.74, (0.000014) |
| lh_parsorbitalis_thickness | 2.44±0.17 | 2.42±0.18 | 2.54±0.17 | F=8.43, (0.000298) |
| lh_parstriangularis_thickness | 2.19±0.14 | 2.21±0.18 | 2.28±0.15 | F=8,38, (0.000314) |
| lh_pericalcarine_thickness | 1.62±0.11 | 1.66±0.14 | 1.64±0.11 | F=3.22, (0.04) |
| lh_postcentral_thickness | 1.89±0.09 | 1.89±0.11 | 1.89±0.09 | F=0.05, (0.96) |
| lh_posteriorcingulate_thickness | 2.34±0.14 | 2.39±0.15 | 2.37±0.14 | F=3.57, (0.03) |
| lh_precentral_thickness | 2.41±0.13 | 2.44±0.13 | 2.45±0.12 | F=1.00, (0.37) |
| lh_precuneus_thickness | 2.31±0.11 | 2.33±0.10 | 2.33±0.11 | F=0.61, (0.55) |
| lh_rostralanteriorcingulate_thickness | 2.60±0.18 | 2.59±0.17 | 2.58±0.17 | F=0.01, (0.99) |
| lh_rostralmiddlefrontal_thickness | 2.14±0.12 | 2.17±0.11 | 2.22±0.12 | F=10.57, (0.000042) |
| lh_superiorfrontal_thickness | 2.58±0.14 | 2.60±0.16 | 2.66±0.14 | F=9.54, (0.000107) |
| lh_superiorparietal_thickness | 2.10±0.09 | 2.12±0.09 | 2.12±0.11 | F=0.67, (0.51) |
| lh_superiortemporal_thickness | 2.50±0.15 | 2.55±0.15 | 2.59±0.14 | F=7.09, (0.001) |
| lh_supramarginal_thickness | 2.18±0.11 | 2.18±0.11 | 2.21±0.12 | F=1.14, (0.32) |
| lh_frontalpole_thickness | 2.56±0.21 | 2.57±0.25 | 2.64±0.23 | F=2.69, (0.07) |
| lh_temporalpole_thickness | 3.51±0.26 | 3.55±0.25 | 3.52±0.24 | F=0.59, (0.56) |
| lh_transversetemporal_thickness | 2.29±0.16 | 2.32±0.20 | 2.37±0.19 | F=3.79, (0.02) |
| lh_insula_thickness | 2.95±0.14 | 2.99±0.13 | 2.99±0.14 | F=1.89, (0.15) |
| rh_bankssts_thickness | 2.23±0.18 | 2.23±0.19 | 2.32±0.18 | F=6.43, (0.002) |
| rh_caudalanteriorcingulate_thickness | 2.23±0.19 | 2.27±0.17 | 2.28±0.19 | F=1.42, (0.24) |
| rh_caudalmiddlefrontal_thickness | 2.38±0.13 | 2.38±0.16 | 2.45±0.14 | F=8.29, (0.000339) |
| rh_cuneus_thickness | 1.91±0.11 | 1.92±0.12 | 1.93±0.12 | F=1.03, (0.36) |
| rh_entorhinal_thickness | 3.41±0.29 | 3.40±0.30 | 3.46±0.29 | F=0.65, (0.52) |
| rh_fusiform_thickness | 2.74±0.13 | 2.78±0.14 | 2.81±0.11 | F=6.22, (0.002) |
| rh_inferiorparietal_thickness | 2.24±0.12 | 2.25±0.13 | 2.25±0.12 | F=0.82, (0.44) |
| rh_inferiortemporal_thickness | 2.63±0.15 | 2.65±0.15 | 2.69±0.11 | F=5.75, (0.004) |
| rh_isthmuscingulate_thickness | 2.22±0.16 | 2.22±0.14 | 2.28±0.14 | F=6.05, (0.003) |
| rh_lateraloccipital_thickness | 2.18±0.11 | 2.21±0.12 | 2.22±0.11 | F=2.81, (0.06) |
| rh_lateralorbitofrontal_thickness | 2.57±0.13 | 2.64±0.14 | 2.68±0.13 | F=19.63, (1.4774E-8) |
| rh_lingual_thickness | 2.02±0.10 | 2.08±0.14 | 2.07±0.12 | F=4.53, (0.01) |
| rh_medialorbitofrontal_thickness | 2.35±0.14 | 2.40±0.15 | 2.46±0.13 | F=15.57, (4.8466E-7) |
| rh_middletemporal_thickness | 2.45±0.14 | 2.44±0.15 | 2.49±0.15 | F=2.59, (0.08) |
| rh_parahippocampal_thickness | 2.60±0.20 | 2.65±0.23 | 2.66±0.21 | F=1.83, (0.16) |
| rh_paracentral_thickness | 2.50±0.13 | 2.49±0.19 | 2.53±0.17 | F=0.81, (0.45) |
| rh_parsopercularis_thickness | 2.37±0.14 | 2.40±0.15 | 2.46±0.14 | F=6.65, (0.002) |
| rh_parsorbitalis_thickness | 2.48±0.18 | 2.54±0.16 | 2.62±0.16 | F=20.09, (1.0063E-8) |
| rh_parstriangularis_thickness | 2.22±0.14 | 2.24±0.15 | 2.33±0.14 | F=14.78, (9.6803E-7) |
| rh_pericalcarine_thickness | 1.64±0.13 | 1.65±0.12 | 1.63±0.12 | F=0.14, (0.87) |
| rh_postcentral_thickness | 1.91±0.10 | 1.91±0.10 | 1.91±0.09 | F=0.00, (1.00) |
| rh_posteriorcingulate_thickness | 2.30±0.12 | 2.35±0.13 | 2.36±0.13 | F=6.65, (0.002) |
| rh_precentral_thickness | 2.42±0.11 | 2.40±0.15 | 2.45±0.13 | F=2.17, (0.12) |
| rh_precuneus_thickness | 2.35±0.11 | 2.37±0.11 | 2.38±0.12 | F=1.36, (0.26) |
| rh_rostralanteriorcingulate_thickness | 2.56±0.19 | 2.59±0.18 | 2.60±0.18 | F=0.76, (0.47) |
| rh_rostralmiddlefrontal_thickness | 2.12±0.12 | 2.16±0.13 | 2.19±0.12 | F=9.42, (0.00012) |
| rh_superiorfrontal_thickness | 2.53±0.14 | 2.56±0.16 | 2.64±0.16 | F=12.28, (0.000009) |
| rh_superiorparietal_thickness | 2.07±0.11 | 2.10±0.11 | 2.09±0.12 | F=0.62, (0.54) |
| rh_superiortemporal_thickness | 2.55±0.15 | 2.57±0.16 | 2.62±0.14 | F=6.28, (0.002) |
| rh_supramarginal_thickness | 2.20±0.12 | 2.19±0.13 | 2.19±0.13 | F=0.25, (0.78) |
| rh_frontalpole_thickness | 2.51±0.25 | 2.56±0.30 | 2.66±0.32 | F=6.19, (0.002) |
| rh_temporalpole_thickness | 3.67±0.22 | 3.72±0.22 | 3.76±0.27 | F=2.01, (0.14) |
| rh_transversetemporal_thickness | 2.30±0.16 | 2.34±0.20 | 2.38±0.19 | F=4.54, (0.01) |
| rh_insula_thickness | 2.96±0.15 | 3.01±0.12 | 3.01±0.16 | F=3.85, (0.02) |

*Note:* M: mean; SD: standard deviation; mm: millimeter; ANCOVA: univariate covariance analysis; AVH: auditory verbal hallucinations; non-AVH: without auditory verbal hallucinations; HC: health control; Bonferroni correction was used for ANCOVA (p＜0.0007).
